# Supplementary material for: Ribonucleotide reductase regulatory subunit M2 (RRM2) as a potential sero-diagnostic biomarker in non-small cell lung cancer
Source: PLoS One. 2023 Sep 12;18(9):e0291461. doi: 10.1371/journal.pone.0291461 (PMC10497127; doi:10.1371/journal.pone.0291461)
Supplement: S1 File — (PDF) [file pone.0291461.s001.pdf]

Supplemental file 1

Four series of matrix files (GSE18842, GSE19188, GSE30219, GSE40791), for a total of 380 NSCLC samples and 224 normal samples, were used to identify DEGs. 258 genes were co-expression, in which 91 genes were clearly up-regulated and 167 genes were clearly down-regulated.

GSE18842 GSE19188 GSE30219 GSE40791	ALL 258	KIF26B RTKN2 ADAM12 CHRDL1 PPP1R14A TPX2 WISP2 PEBP4 IGF2BP3 CCNB1 SLC6A4 HMGB3 KCNK3 DSP NOSTRIN SYNPO2 CXCL13 GIMAP8 SUSD2 FRY BTNL9 VEPH1 AKR1B10 GINS1 TEK COL1A1 DACH1 VGLL3 ADAMDEC1 ANLN UCHL1 BIRC5 FAM150B ZNF385B EZH2 CDK1 GDF10 FNDC1 DNASE1L3 CHEK1 KIF11 CD36 C14orf132 SPTBN1 LRRK2 PPBP PTPRB ACADL AQP4 PIR-FIGF///FIGF NEBL ITGA8 MT1M TNNC1 HLF AURKA MCEMP1 KIF14 FHL1 MAD2L1 CPB2 SORBS2 ARNTL2 ABCA8 AOC3 ADRB1 TCF21 GLDN UPK3B KIF4A ASPA CCL15-CCL14///CCL14 EDNRB TMPRSS4 HS6ST2 MYCT1 KANK3 SPP1 SFTPD STX11 PLAC9 MYH11 C1QTNF7 KIF2C OLR1 AGER RGCC VWF ABI3BP AGTR1 WIF1 TPPP3 TYMS FAM72A///FAM72D ///FAM72B///FAM72C CDCA7 FLJ35700 MELK GPX2 CLIC5 CDC20 ADRB2 ZWINT PLEKHH2 NDC80 FHL5 GREM1 CCNA2 GTSE1 C7 BUB1 NUF2 PBK COCH KRT6A PDK4 GPM6A FILIP1 MMP1 MMP12 ANGPT1 UBE2T DUXAP10 SLC2A1 TMEM100 ECT2 CACNA2D2 KIF23 DEPDC1 HBA2///HBA1 TPSAB1 EMCN ADARB1 LMO7 GPIHBP1 MFAP4 KCNT2 ASPM NCKAP5 OGN SCARA5 IGSF10 LINC00673///LINC00511 RBP4 CDCA3 SCGB1A1 CDO1 CA4 SDPR ID4 EXOSC7///CLEC3B PLA2G1B ACKR1 ANOS1 SORBS1 LIFR UBE2C STXBP6 CCNB2 LYVE1 PRC1 ADAMTS8 SLC1A1 SRD5A1 PSAT1 PTPN21 TNXB///TNXA LIMCH1 LEPROT///LEPR TPSB2 CEP55 SPOCK2 CLU RRM2 SLC35F6///CENPA TOP2A LDB2 CPA3 ROBO4 GJB2 ERO1A WDR72 CALCRL CAV1 RAI2 JAM2 FOXF1 S100A2 ANKRD29 EP300-AS1 INMT ADIRF CELF2 HBB TFAP2A RHOJ BUB1B FAM189A2 DLGAP5 LRRN3 HJURP SCN7A FMO2 MYZAP SLC39A8 RBMS3 SFTPC IL33 SULF1 TGFBR3 HHIP ADH1B FERMT1 ARHGAP6 LPL HMMR LINC00968 FABP4 FCN3 DLC1 LINC00312 CCDC85A FAM107A NMU CCBE1 TPSB2///TPSAB1 PGM5 GPX3 KIF20A CAV2 FOSB LOC101926959 MARCO PIP5K1B

		COL10A1 SFTPA2///SFTPA1 GPR87 COL11A1 SLIT2 CX3CR1 CTHRC1 UHRF1 KIAA0101 MAMDC2 PCOLCE2 TTK CDKN3 SCG5 CLDN18 NCAPG C2orf40 COL6A6 CFD NEK2 GKN2 CYP4B1 CENPF NUSAP1 AFF3
	DOWN 167	HBA2///HBA1 TPSAB1 RTKN2 EMCN ADARB1 LMO7 CHRDL1 PPP1R14A WISP2 GPIHBP1 KCNT2 MFAP4 PEBP4 SLC6A4 KCNK3 NOSTRIN SYNPO2 NCKAP5 GIMAP8 OGN SUSD2 FRY SCARA5 BTNL9 VEPH1 IGSF10 RBP4 SCGB1A1 CDO1 CA4 SDPR TEK ID4 EXOSC7///CLEC3B DACH1 PLA2G1B VGLL3 FAM150B ANOS1 ACKR1 SORBS1 LIFR STXBP6 ZNF385B LYVE1 ADAMTS8 SLC1A1 PTPN21 TNXB///TNXA GDF10 LIMCH1 LEPROT///LEPR DNASE1L3 TPSB2 SPOCK2 CD36 CLU C14orf132 LDB2 CPA3 ROBO4 SPTBN1 CALCRL CAV1 RAI2 LRRK2 PPBP JAM2 PTPRB FOXF1 ACADL ANKRD29 AQP4 PIR-FIGF///FIGF NEBL ITGA8 MT1M TNNC1 HLF EP300-AS1 INMT ADIRF MCEMP1 CELF2 HBB FHL1 RHOJ CPB2 FAM189A2 SORBS2 LRRN3 SCN7A FMO2 ABCA8 MYZAP SLC39A8 AOC3 RBMS3 SFTPC ADRB1 IL33 TCF21 GLDN TGFBR3 HHIP ADH1B UPK3B ARHGAP6 LPL LINC00968 ASPA CCL15-CCL14///CCL14 FABP4 EDNRB FCN3 MYCT1 KANK3 DLC1 SFTPD STX11 LINC00312 PLAC9 FAM107A CCDC85A MYH11 CCBE1 PGM5 TPSB2///TPSAB1 C1QTNF7 GPX3 OLR1 AGER FOSB CAV2 RGCC LOC101926959 VWF MARCO PIP5K1B ABI3BP SFTPA2///SFTPA1 AGTR1 WIF1 TPPP3 FLJ35700 CLIC5 SLIT2 CX3CR1 ADRB2 PLEKHH2 FHL5 MAMDC2 PCOLCE2 C7 CLDN18 C2orf40 PDK4 GPM6A COL6A6 FILIP1 CFD GKN2 ANGPT1 CYP4B1 TMEM100 CACNA2D2 AFF3
	UP 91	KIF26B ADAM12 TPX2 IGF2BP3 CCNB1 SULF1 HMGB3 DSP FERMT1 ASPM HMMR CXCL13 KIF4A AKR1B10 LINC00673///LINC00511 CDCA3 GINS1 TMPRSS4 HS6ST2 SPP1 COL1A1 NMU ADAMDEC1 ANLN KIF2C UCHL1 BIRC5 KIF20A UBE2C EZH2 COL10A1 CCNB2 PRC1 SRD5A1 CDK1 PSAT1 GPR87 FAM72A///FAM72D///FAM72B ///FAM72C TYMS CDCA7 FNDC1 CHEK1 MELK COL11A1 KIF11 GPX2 CEP55 CDC20 CTHRC1 UHRF1 RRM2 ZWINT SLC35F6///CENPA NDC80 TOP2A KIAA0101 GJB2 ERO1A WDR72 GREM1 CCNA2 TTK GTSE1 CDKN3 BUB1 SCG5 NUF2 PBK COCH NCAPG S100A2 KRT6A MMP1 NEK2 MMP12 AURKA UBE2T SLC2A1 DUXAP10 CENPF NUSAP1 KIF14 TFAP2A BUB1B MAD2L1 DLGAP5 ECT2 DEPDC1 KIF23 HJURP ARNTL2
