# Supplementary material for: Ribonucleotide reductase regulatory subunit M2 (RRM2) as a potential sero-diagnostic biomarker in non-small cell lung cancer
Source: PLoS One. 2023 Sep 12;18(9):e0291461. doi: 10.1371/journal.pone.0291461 (PMC10497127; doi:10.1371/journal.pone.0291461)
Supplement: S2 File — (PDF) [file pone.0291461.s002.pdf]

Supplemental file 2

The expression levels and the survival analyses of hub genes (RRM2, KIF11, UBE2C, MAD2L1, KIF20A, CDK1, CCNB2, BUB1, CCNA2, BUB1B) were shown in **Fig. S1-S10**.

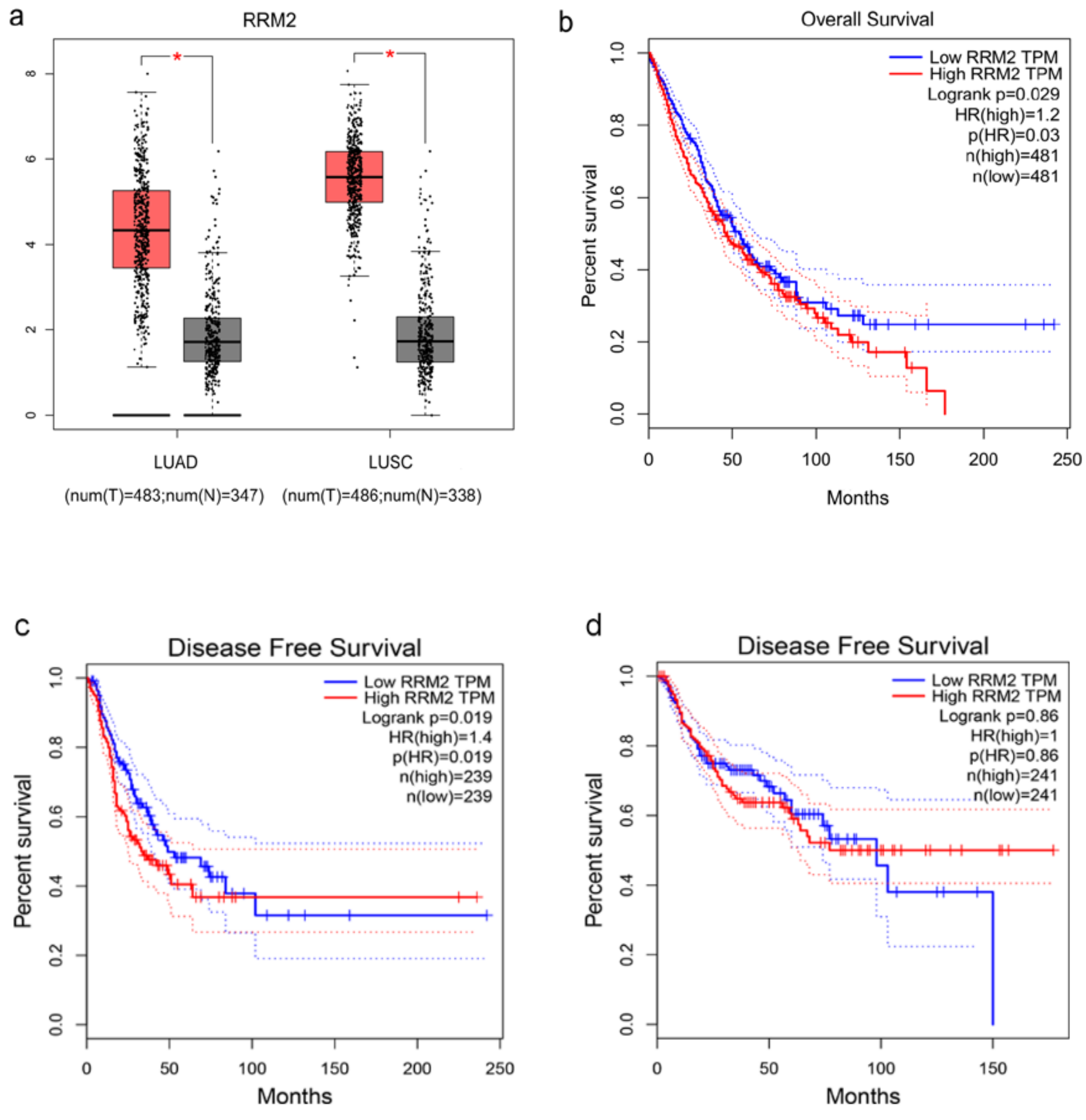

Fig. S1 The expression level and survival analysis of RRM2.
a: expression level analysis, **b:** overall survival analysis, **c:** disease-free survival in LUAD, **d**: disease-free survival in LUSC

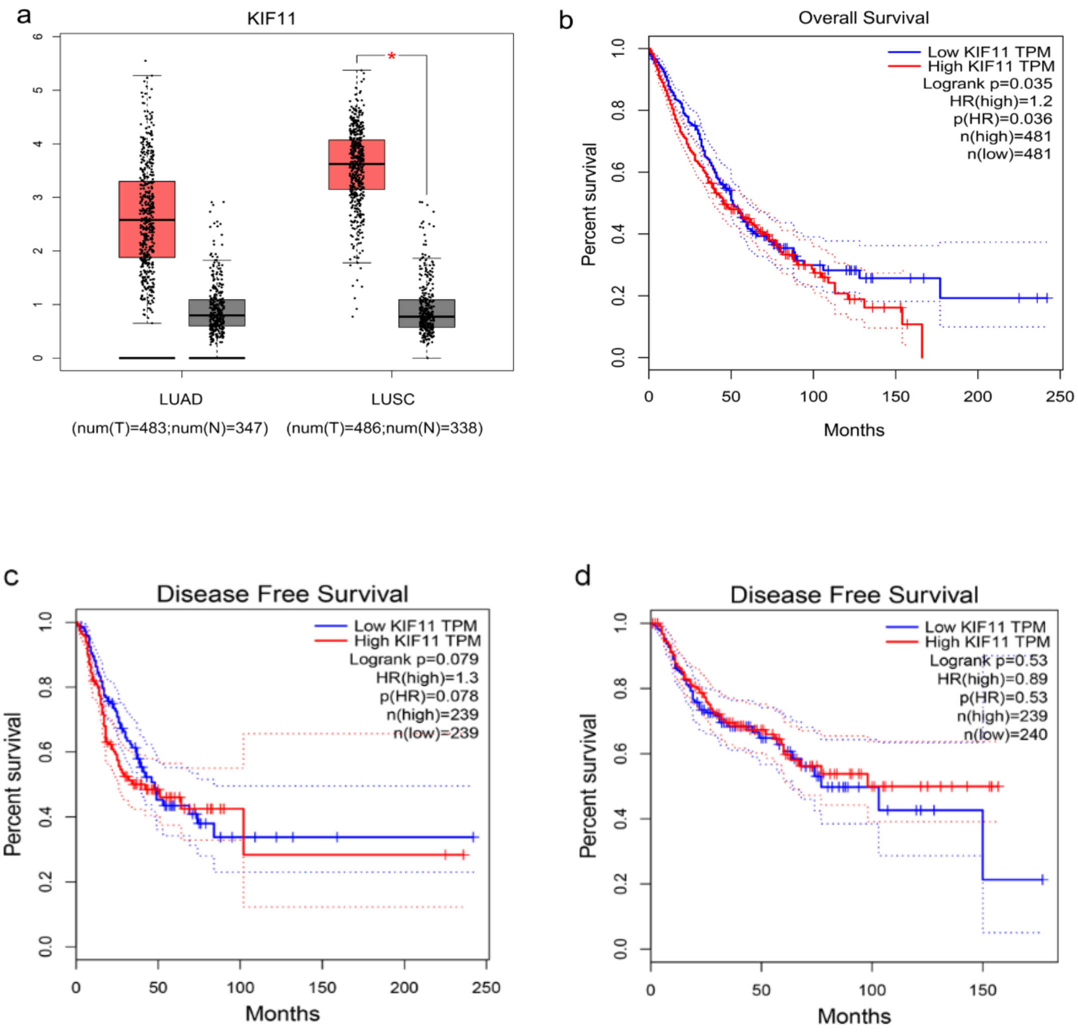

Fig. S2 The expression level and survival analysis of KIF11.

a: expression level analysis, **b:** overall survival analysis, **c:** disease-free survival in LUAD, **d**: disease-free survival in LUSC

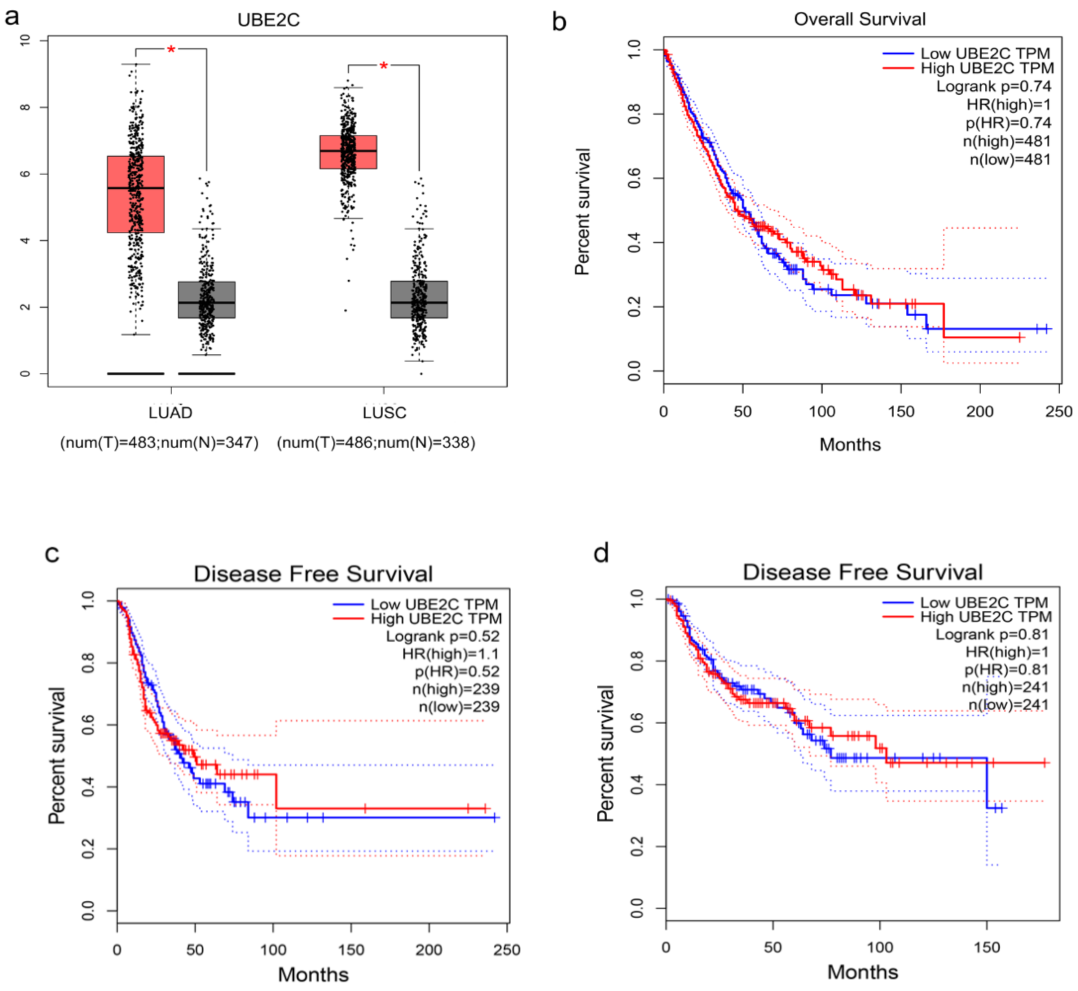

Fig. S3 The expression level and survival analysis of UBE2C.
a: expression level analysis, **b:** overall survival analysis, **c:** disease-free survival in LUAD, **d**: disease-free survival in LUSC

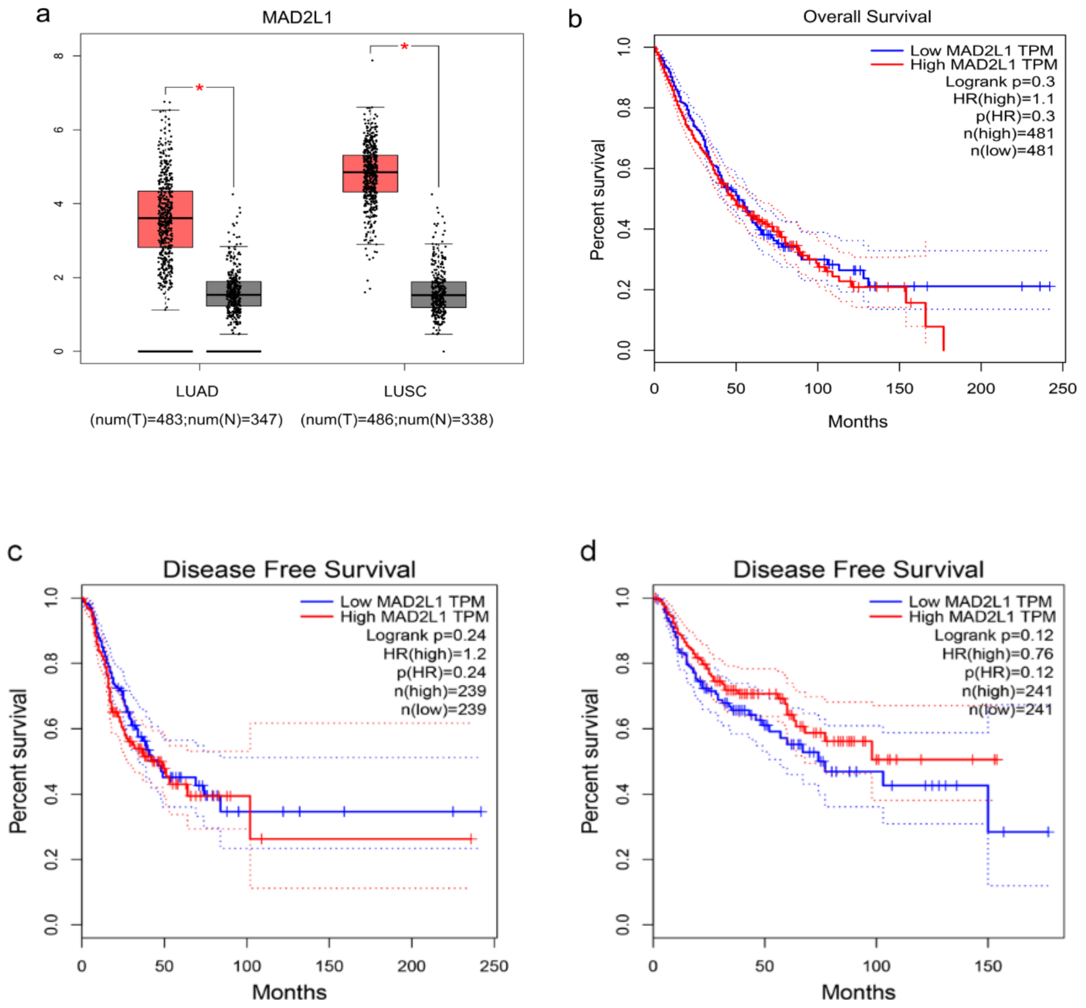

Fig. S4 The expression level and survival analysis of MAD2L1.
a: expression level analysis, **b:** overall survival analysis, **c:** disease-free survival in LUAD, **d**: disease-free survival in LUSC

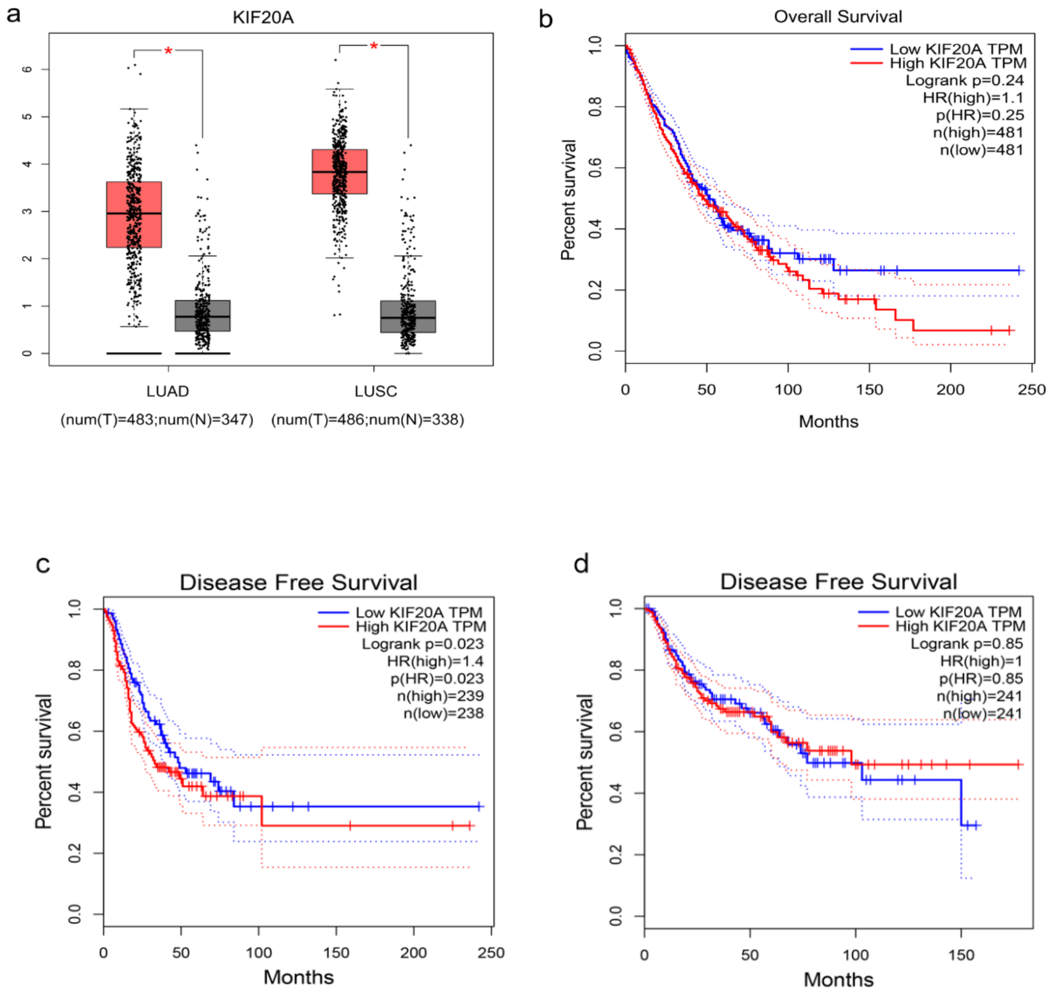

Fig. S5 The expression level and survival analysis of KIF20A.
a: expression level analysis, **b:** overall survival analysis, **c:** disease-free survival in LUAD, **d**: disease-free survival in LUSC

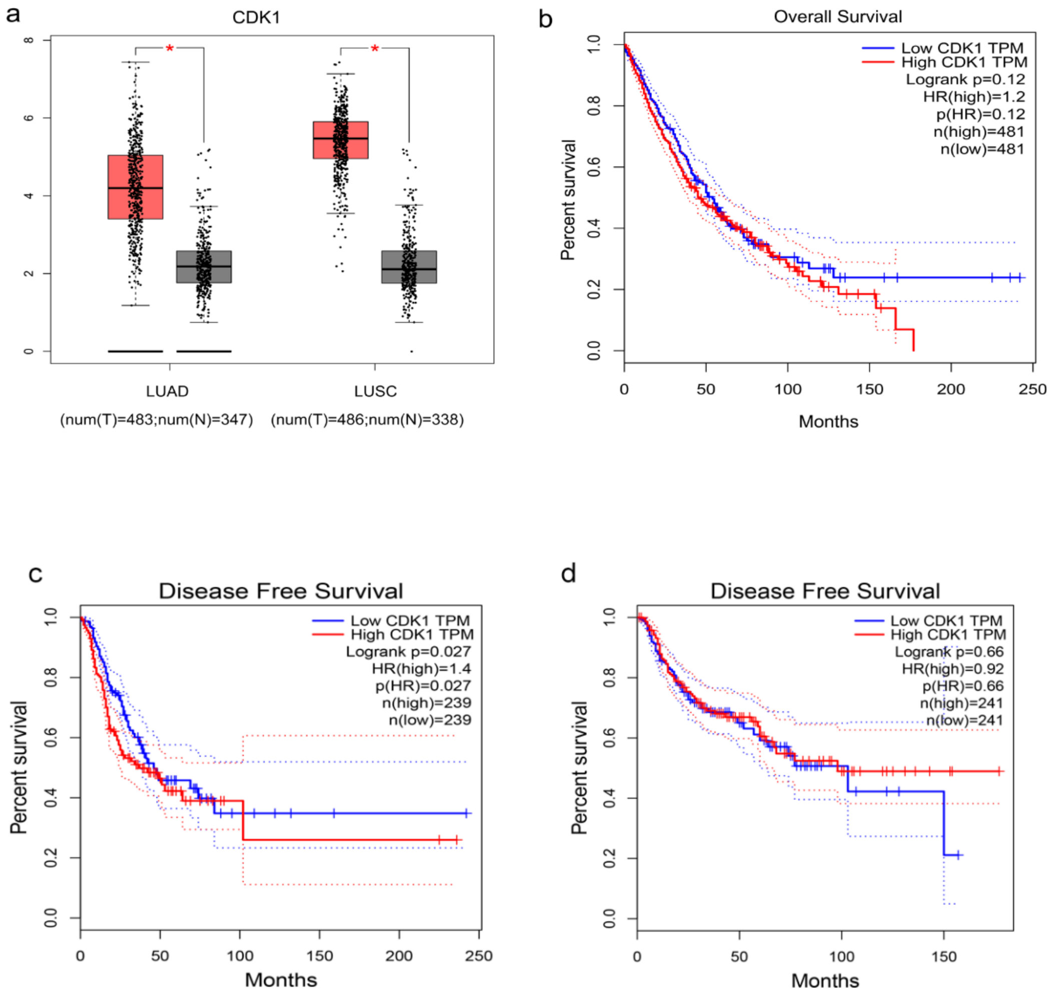

Fig. S6 The expression level and survival analysis of CDK1.
a: expression level analysis, **b:** overall survival analysis, **c:** disease-free survival in LUAD, **d**: disease-free survival in LUSC

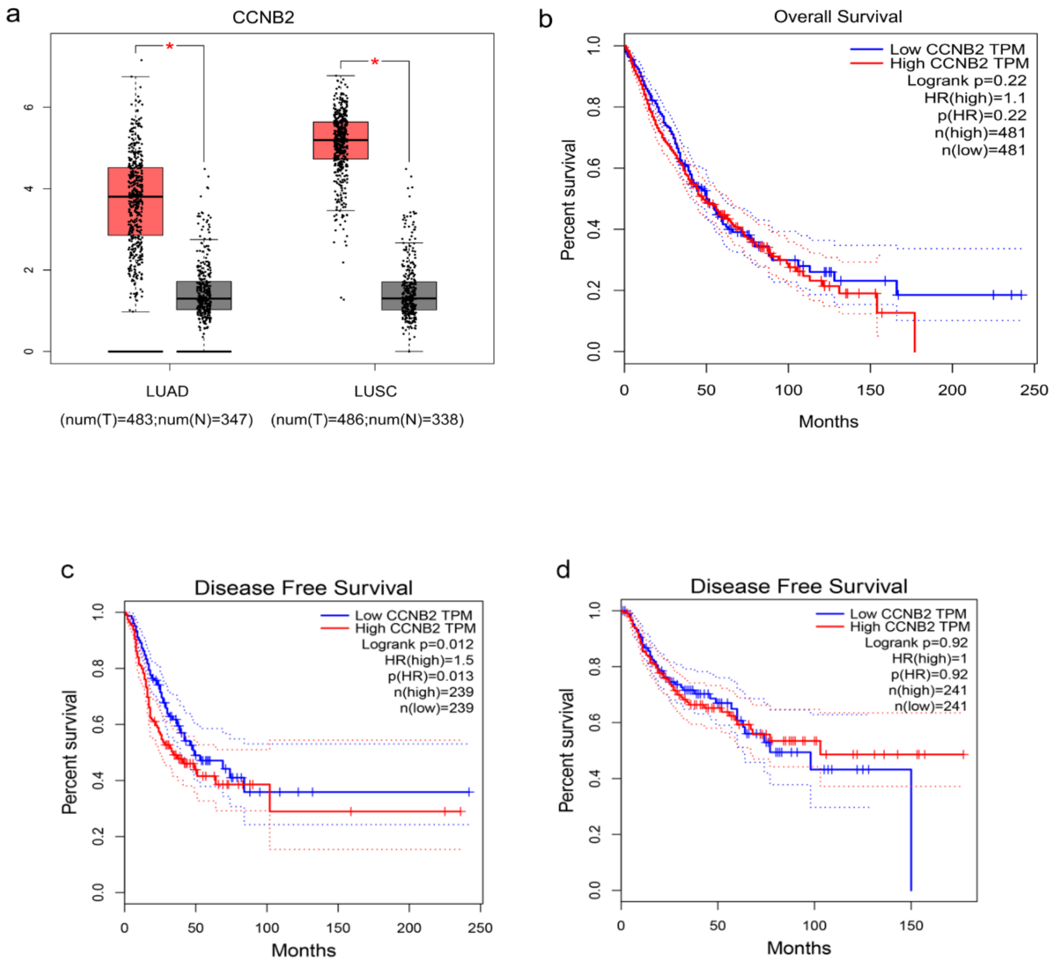

Fig. S7 The expression level and survival analysis of CCNB2.
a: expression level analysis, **b:** overall survival analysis, **c:** disease-free survival in LUAD, **d**: disease-free survival in LUSC

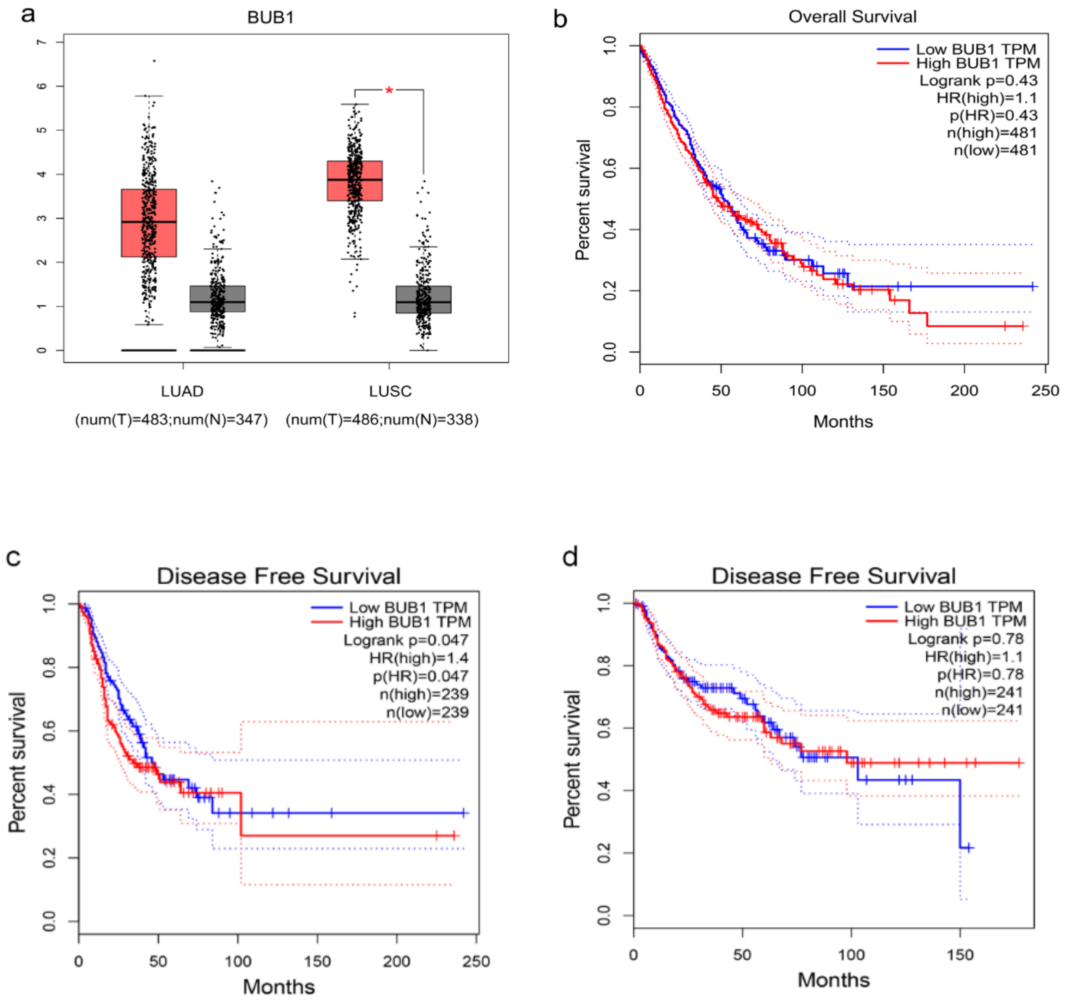

Fig. S8 The expression level and survival analysis of BUB1.
a: expression level analysis, **b:** overall survival analysis, **c:** disease-free survival in LUAD, **d**: disease-free survival in LUSC

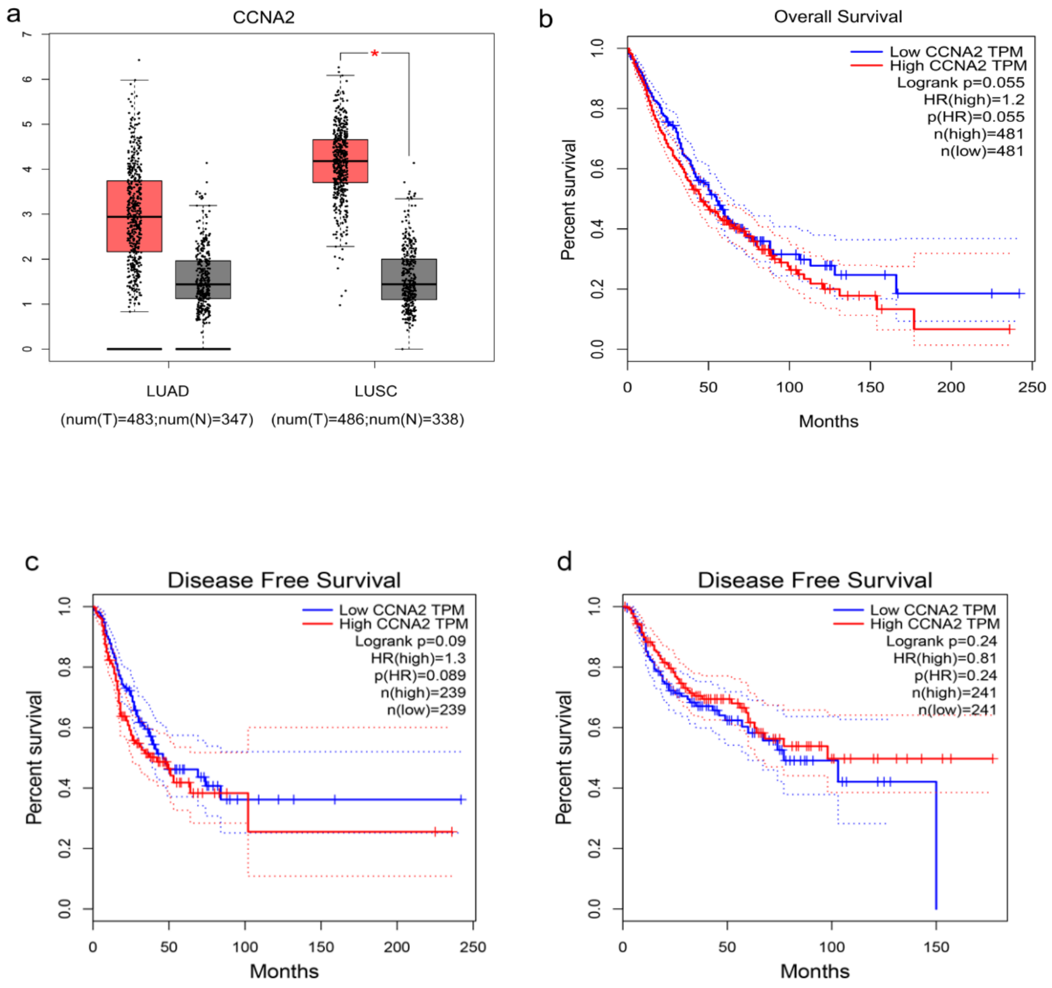

Fig. S9 The expression level and survival analysis of CCNA2.
a: expression level analysis, **b:** overall survival analysis, **c:** disease-free survival in LUAD, **d**: disease-free survival in LUSC

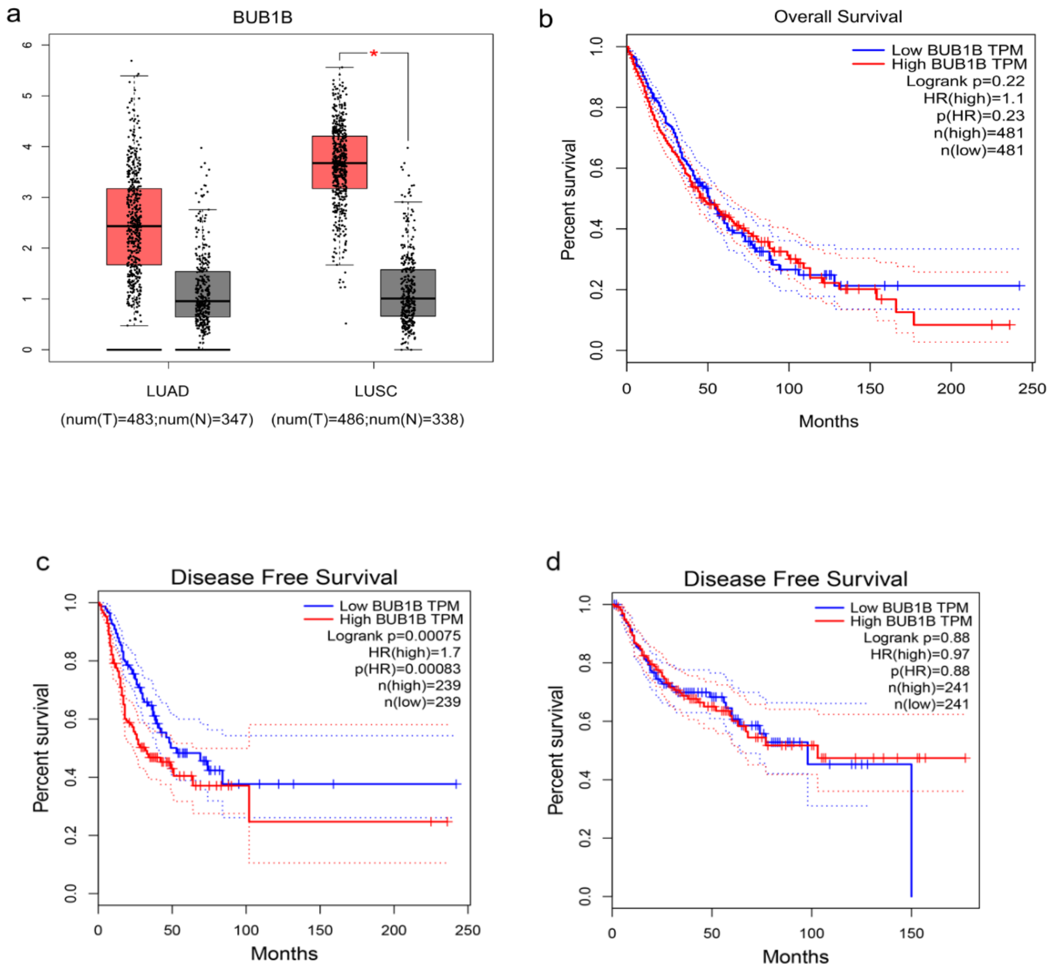

Fig. S10 The expression level and survival analysis of BUB1B.
a: expression level analysis, **b:** overall survival analysis, **c:** disease-free survival in LUAD, **d**: disease-free survival in LUSC
