# Supplementary material for: Ribonucleotide reductase regulatory subunit M2 (RRM2) as a potential sero-diagnostic biomarker in non-small cell lung cancer
Source: PLoS One. 2023 Sep 12;18(9):e0291461. doi: 10.1371/journal.pone.0291461 (PMC10497127; doi:10.1371/journal.pone.0291461)
Supplement: S3 File — (PDF) [file pone.0291461.s003.pdf]

Supplemental file 3

KEGG pathway analysis of hub genes

Category Term	Gene	*P* Value
Cell cycle	CDK1 MAD2L1 CCNB2 BUB1 BUB1B CCNA2	1.19103^{E-11}
Oocyte meiosis	CDK1 MAD2L1 CCNB2 BUB1	3.52519^{E-07}
p53 signaling pathway	RRM2 CDK1 CCNB2	7.88349^{E-07}
Progesterone-mediated oocyte maturation	CDK1 MAD2L1 CCNB2 BUB1 CCNA2	2.91491^{E-06}
Viral carcinogenesis	CDK1 CCNA2	1.77696^{E-02}
HTLV-I infection	MAD2L1 BUB1B	3.11221^{E-02}
