# Supplementary material for: Ribonucleotide reductase regulatory subunit M2 (RRM2) as a potential sero-diagnostic biomarker in non-small cell lung cancer
Source: PLoS One. 2023 Sep 12;18(9):e0291461. doi: 10.1371/journal.pone.0291461 (PMC10497127; doi:10.1371/journal.pone.0291461)
Supplement: S4 File — (PDF) [file pone.0291461.s004.pdf]

The mRNA relative expression of RRM[illegible]

	TNM stage		GAPDH		RRM2		
			Ct	Ct average	Ct	Ct average	△Ct
		Tumor01-1	19.28		20.24		
	I	Tumor01-2	17.75	18.34	19.36	19.91	1.57
		Tumor01-3	17.98		20.13		
		Tumor02-1	18.75		20.14		
	II	Tumor02-2	19.03	19.09	20.79	20.52	1.43
		Tumor02-3	19.49		20.62		
		Tumor03-1	18.48		20.18		
	III	Tumor03-2	18.78	18.62	20.38	20.07	1.46
		Tumor03-3	18.59		19.66		
		Tumor04-1	18.23		20.25		
	III	Tumor04-2	18.52	18.69	20.96	20.42	1.72
		Tumor04-3	19.33		20.04		
		Tumor05-1	18.21		20.08		
	IV	Tumor05-2	18.22	18.14	20.19	20.11	1.97
		Tumor05-3	17.98		20.06		
		Tumor06-1	19.06		22.21		
	IV	Tumor06-2	18.96	19.04	22.32	22.16	3.11
		Tumor06-3	19.11		21.94		
		Tumor07-1	20.18		21.99		
	IV	Tumor07-2	20.13	20.12	21.88	21.95	1.83
		Tumor07-3	20.04		21.98		
		Tumor08-1	19.14		20.6		
	II	Tumor08-2	19.01	19.02	20.05	20.47	1.45
		Tumor08-3	18.92		20.76		
		Tumor09-1	19.68		20.22		
	I	Tumor09-2	18.9	19.16	20.18	20.25	1.08
LUSC		Tumor09-3	18.91		20.34		
		Tumor10-1	19.51		21.3		
	IV	Tumor10-2	19.56	19.68	21.7	21.50	1.82
		Tumor10-3	19.98		21.5		
		Tumor11-1	19.62		21.62		
	IV	Tumor11-2	19.95	19.77	21.76	21.74	1.98
		Tumor11-3	19.73		21.85		
		Tumor12-1	18.94		20.42		
	III	Tumor12-2	19.11	18.96	20.75	20.82	1.86
		Tumor12-3	18.82		21.29		
		Tumor13-1	18.46		20.66		
	IV	Tumor13-2	18.97	18.60	20.97	20.75	2.15
		Tumor13-3	18.37		20.63		
		Tumor14-1	19.5		21.59		
	I	Tumor14-2	20.02	19.73	21.89	21.71	1.98
		Tumor14-3	19.66		21.65		
		Tumor15-1	19.34		21.16		
	III	Tumor15-2	19.22	19.39	21.3	21.13	1.74
		Tumor15-3	19.6		20.93		
		Tumor16-1	19.86		20.38		
	II	Tumor16-2	19.23	19.28	20.5	20.48	1.20
		Tumor16-3	18.74		20.56		
		Tumor17-1	19.24		21.18		
	IV	Tumor17-2	18.28	18.52	21.5	21.42	2.90

		Tumor17-3	18.05		21.59		
		Tumor18-1	19.49		20.27		
	II	Tumor18-2	18.32	18.90	19.37	20.06	1.16
		Tumor18-3	18.89		20.53		
		Tumor19-1	19.49		20		
	I	Tumor19-2	17.99	18.53	19.08	19.31	0.78
		Tumor19-3	18.12		18.86		
		Tumor20-1	19.43		20.43		
	I	Tumor20-2	18.39	18.78	19.26	19.62	0.84
		Tumor20-3	18.51		19.17		
		Tumor21-1	19.16		19.78		
	II	Tumor21-2	18.04	18.85	19.3	19.63	0.78
		Tumor21-3	19.34		19.8		
		Tumor22-1	18.03		19.14		
	III	Tumor22-2	19.68	18.65	20.83	19.79	1.14
		Tumor22-3	18.23		19.39		
		Tumor23-1	18.58		20.55		
	IV	Tumor23-2	18.99	18.77	20.83	20.58	1.81
		Tumor23-3	18.73		20.35		
		Tumor24-1	18.86		20.51		
	II	Tumor24-2	19.11	19.06	20.68	20.59	1.53
		Tumor24-3	19.2		20.58		
LUAD		Tumor25-1	19.49		20.94		
	II	Tumor25-2	19.44	19.51	20.66	20.64	1.13
		Tumor25-3	19.59		20.32		
		Tumor26-1	18.83		20.95		
	III	Tumor26-2	18.93	18.80	20.69	20.73	1.93
		Tumor26-3	18.64		20.56		
		Tumor27-1	18.58		19.15		
	I	Tumor27-2	18.99	18.77	19.83	19.44	0.68
		Tumor27-3	18.73		19.35		
		Tumor28-1	18.86		19.51		
	I	Tumor28-2	19.51	19.19	20.38	20.16	0.97
		Tumor28-3	19.2		20.58		
		Tumor29-1	19.49		20.94		
	II	Tumor29-2	19.44	19.34	20.66	20.81	1.47
		Tumor29-3	19.09		20.82		
		Tumor30-1	18.83		19.29		
	III	Tumor30-2	18.73	18.73	19.49	19.75	1.01
		Tumor30-3	18.64		20.46		

2 in NSCLC tissues and adjacent normal tissues

	GAPDH		RRM2		
	Ct	Ct average	Ct	Ct average	△Ct
Adjacent01-1	18.89		19.52		
Adjacent01-2	18.31	18.73	19.83	19.67	0.94
Adjacent01-3	18.98		19.65		
Adjacent02-1	18.99		20.26		
Adjacent02-2	19.67	19.54	20.37	20.31	0.76
Adjacent02-3	19.97		20.29		
Adjacent03-1	21.15		22.67		
Adjacent03-2	20.94	21.11	21.39	21.86	0.75
Adjacent03-3	21.24		21.52		
Adjacent04-1	19.56		20.28		
Adjacent04-2	19.17	19.34	20.77	20.39	1.05
Adjacent04-3	19.29		20.13		
Adjacent05-1	18.92		19.25		
Adjacent05-2	19.11	19.07	19.59	19.37	0.30
Adjacent05-3	19.18		19.28		
Adjacent06-1	19.96		20.82		
Adjacent06-2	21.05	20.67	21.24	21.32	0.64
Adjacent06-3	21.01		21.89		
Adjacent07-1	19.47		20.06		
Adjacent07-2	19.28	19.36	19.88	20.01	0.65
Adjacent07-3	19.32		20.08		
Adjacent08-1	19.52		20.01		
Adjacent08-2	19.06	19.53	20.19	20.40	0.87
Adjacent08-3	20.01		21.01		
Adjacent09-1	19.59		20.32		
Adjacent09-2	19.61	19.54	20.08	20.25	0.71
Adjacent09-3	19.43		20.36		
Adjacent10-1	19.87		21.53		
Adjacent10-2	19.55	19.89	20.35	21.07	1.18
Adjacent10-3	20.26		21.34		
Adjacent11-1	20.03		20.75		
Adjacent11-2	19.83	20.01	20.95	20.77	0.76
Adjacent11-3	20.17		20.61		
Adjacent12-1	19.92		21.08		
Adjacent12-2	20.19	20.02	20.57	20.87	0.85
Adjacent12-3	19.94		20.96		
Adjacent13-1	19.81		20.01		
Adjacent13-2	19.94	19.65	20.32	20.18	0.52
Adjacent13-3	19.21		20.2		
Adjacent14-1	20.17		21.09		
Adjacent14-2	19.54	20.12	21.01	21.03	0.91
Adjacent14-3	20.64		20.98		
Adjacent15-1	21.18		21.8		
Adjacent15-2	21.99	21.70	22.49	22.21	0.51
Adjacent15-3	21.94		22.34		
Adjacent16-1	20.82		21.45		
Adjacent16-2	19.99	20.53	20.25	21.19	0.66
Adjacent16-3	20.77		21.87		
Adjacent17-1	19.57		20.39		
Adjacent17-2	19.86	19.69	20.85	20.59	0.90

Adjacent17-3	19.63		20.53		
Adjacent18-1	19.92		20.8		
Adjacent18-2	19.96	20.01	20.11	20.55	0.54
Adjacent18-3	20.14		20.74		
Adjacent19-1	20.93		21.29		
Adjacent19-2	19.93	20.25	20.64	20.90	0.65
Adjacent19-3	19.88		20.76		
Adjacent20-1	19.45		19.72		
Adjacent20-2	20.61	20.40	20.79	20.61	0.21
Adjacent20-3	21.14		21.32		
Adjacent21-1	20.01		20.35		
Adjacent21-2	19.9	19.94	20.78	20.66	0.72
Adjacent21-3	19.92		20.86		
Adjacent22-1	18.94		19.24		
Adjacent22-2	18.98	18.71	19.55	19.15	0.44
Adjacent22-3	18.21		18.65		
Adjacent23-1	20.83		21.41		
Adjacent23-2	21.09	20.96	21.25	21.39	0.44
Adjacent23-3	20.95		21.52		
Adjacent24-1	20.18		20.54		
Adjacent24-2	21.07	20.60	21.06	20.91	0.31
Adjacent24-3	20.54		21.13		
Adjacent25-1	20.44		20.99		
Adjacent25-2	20.65	20.46	21.6	21.05	0.58
Adjacent25-3	20.3		20.55		
Adjacent26-1	20.76		21.19		
Adjacent26-2	20.63	20.65	21.41	21.06	0.40
Adjacent26-3	20.57		20.57		
Adjacent27-1	20.53		20.81		
Adjacent27-2	20.09	20.36	20.55	20.66	0.30
Adjacent27-3	20.45		20.62		
Adjacent28-1	20.18		20.94		
Adjacent28-2	20.07	20.26	21.06	21.04	0.78
Adjacent28-3	20.54		21.13		
Adjacent29-1	20.64		21.39		
Adjacent29-2	20.65	20.71	21.26	21.30	0.59
Adjacent29-3	20.83		21.25		
Adjacent30-1	19.76		21.19		
Adjacent30-2	20.63	20.32	21.49	21.15	0.83
Adjacent30-3	20.57		20.77		
