# Supplementary material for: Ribonucleotide reductase regulatory subunit M2 (RRM2) as a potential sero-diagnostic biomarker in non-small cell lung cancer
Source: PLoS One. 2023 Sep 12;18(9):e0291461. doi: 10.1371/journal.pone.0291461 (PMC10497127; doi:10.1371/journal.pone.0291461)
Supplement: S5 File — (PDF) [file pone.0291461.s005.pdf]

Male=1 Fe	Age	rcinoma=0	分期1	T	N	M	TNM stage	=II、33=II	EA(0-5)ng/	RA(0-3.3)n	(0-16.3)ng	(25.3-69.2	RRM2	Group	3<22≤5cm	Tumor size	tastasis	astasis 11I)	Advance 22=(III + IV)
1	57	1	T4N2M0	4	2	0	IV	44	3.12	1.47	42.99	557.6	143.82	1	44	22	22	11	22
1	55	0	T2N2M0	2	2	0	III	33	12.39	9.45	214.5	382.6	161.33	1	22	22	22	11	22
1	66	0	T2N0M1a	2	0	1a	IVA	44	41.98	2.56	18.55	135.9	152.85	1	22	22	11	22	22
1	56	0	T1N3M1	1	3	1	III	33	2.33	1.26	17.2	109.4	156.91	1	11	11	22	22	22
1	69	0	TxN0M1a	x	0	1a	IVA	44	9.43	4.11	37.24	101.8	150.55	1	XX		11	22	22
1	66	0	T2N0M1	2	0	1	IVA	44	41.46	4.63	10.21	101.5	167.39	1	22	22	11	22	22
1	66	0	T4N2M0	4	2	0	III	33	11.47	5.65	19.55	99.23	157.82	1	44	22	22	11	22
0	71	0	T4NXM0	4	x	0	III	33	144.6	4.94	23.2	99.1	149.01	1	44	22		11	22
1	63	1	T3N1M0	3	1	0	IV	44	11.6	2.13	12.25	98.23	141.86	1	33	22	22	11	22
1	55	0	T2N2M1	2	2	1	IV	44	1.47	2.99	18.23	89.28	136.11	1	22	22	22	22	22
1	58	0	T4N2M1	4	2	1	IVb	44	3.32	5.19	16.23	88.96	152.18	1	44	22	22	22	22
1	66	0	T3N2M0	3	2	0	IIIB	33	4.51	10.5	34.56	85.15	148.41	1	33	22	22	11	22
1	62	0	TxNxM1	x	x	1	IV	44	3.57	2.37	16.42	79.9	132.55	1	XX			22	22
1	67	1	T3N2M0	3	2	0	III	33	2.71	1.76	18.9	76.82	68.05	1	33	22	22	11	22
1	67	0	T3N1M0	3	1	0	IIIA	33	6.21	3.12	17.11	74.83	156.87	1	33	22	22	11	22
1	80	1	T3N2M1	3	2	1	IVA	44	5	3.61	17.01	74.71	164.62	1	33	22	22	22	22
1	64	1	T4N2M1	4	2	1	IV	44	4.57	56.98	15.48	74.66	174.32	1	44	22	22	22	22
0	52	0	T2N2M1	2	2	1	IV	44	7.28	2.86	90.62	74.33	158.11	1	22	22	22	22	22
1	71	0	T2N0M0	2	0	0	I	11	2.58	6.16	26.14	73.49	81.49	1	22	22	11	11	11
0	53	1	T1N3M1	1	3	1	II	22	2.55	2.39	43.62	72.87	143.78	1	11	11	22	22	11
0	58	0	T1N2M1	1	2	1	IVB	44	43.76	9.15	295.1	70.31	170.63	1	11	11	22	22	22
1	67	0	T3N1M0	3	1	0	IIIA	33	5.96	2.11	12.18	69.81	139.80	1	33	22	22	11	22
1	82	0	T3N3M1	3	3	1	III	33	6.8	7.58	9.53	69.3	167.92	1	33	22	22	22	22
0	65	0	T4N2M1	4	2	1	IVB	44	5.16	3.68	25.24	68.67	161.04	1	44	22	22	22	22
0	55	0	T4N2M1c	4	2	1c	IVB	44	60.58	16.37	20.62	67.04	170.78	1	44	22	22	22	22
1	74	1	T1N0M0	1	0	0	IA	11	5.66	2.22	13.25	66.8	161.07	1	11	11	11	11	11
0	52	0	T2N2M1	2	2	1	IV	44	6.11	2.35	26.68	65.89	171.28	1	22	22	22	22	22
1	62	0	T4N1M1	4	1	1	IVB	44	4.13	2.57	10.7	65.6	125.90	1	44	22	22	22	22
0	82	0	T4N2M1	4	2	1	IVB	44	26.1	52.7	40.59	65.46	174.29	1	44	22	22	22	22
0	63	1	T4N0M0	4	0	0	IIIa	33	1.72	3.29	12.38	64.45	168.42	1	44	22	11	11	22
1	78	1	T3N0M0	3	0	0	IIB	22	5.26	1.35	14.45	64.27	80.57	1	33	22	11	11	11
1	69	1	T1N2M0	1	2	0	IIIa	33	1.95	1.54	29.92	64.22	138.81	1	11	11	22	11	22
1	71	1	T3N1M0	3	1	0	IIIA	33	3.8	2.66	15.51	63.33	135.52	1	33	22	22	11	22
0	79	0	T4N3M1c	4	3	1c	IVB	44	7.26	8.42	12.92	61.95	136.36	1	44	22	22	22	22
1	71	1	T3N1M0	3	1	0	IIIa	33	2.85	2.99	19.53	60.83	129.37	1	33	22	22	11	22
0	67	0	T2aN0M0	2a	0	0	Ib	11	6.12	1.66	15.24	59.16	129.84	1	22	22	11	11	11
1	68	0	T2N3M1	2	3	1	II	22	1.06	2.07	19.65	58.69	102.59	1	22	22	22	22	11
0	50	0	T2N2M0	2	2	0	IIIA	33	25.69	1.91	19.48	58.36	165.51	1	22	22	22	11	22
1	75	1	T4NXMX	4	x	x	III	33	6.28	1.89	20.13	57.01	120.40	1	44	22			22
1	62	0	T2N3M1c	2	3	1c	IVB	44	20.3	2.63	20.64	56.41	174.41	1	22	22	22	22	22
1	70	0	T2N3M1	2	3	1	IVb	44	2.54	10.73	23.86	56.35	174.41	1	22	22	22	22	22
1	57	0	T4N2M1	4	2	1	IVB	44	2.91	2.49	17.44	55.72	154.76	1	44	22	22	22	22
1	72	0	TxN0M1a	x	0	1a	IVA	44	39.42	4.43	20.54	55.57	172.00	1	XX		11	22	22
1	70	0	T2N0M1	2	0	1	IVB	44	306.2	1.88	39.03	53.76	89.56	1	22	22	11	22	22
1	79	0	T2N3M0	2	3	0	IIIB	33	10.48	4.71	18.3	53.58	174.41	1	22	22	22	11	22
1	71	0	T1N0M0	1	0	0	IA	11	2.89	3.39	13.79	53.56	115.70	1	11	11	11	11	11
1	77	0	T4NXM1b	4	x	1b	IV	44	3.2	3.78	20.18	53.2	93.88	1	44	22		22	22
0	41	0	T2aN2M0	2a	2	0	IIIA	33	8.04	13.9	370	53.04	173.29	1	22	22	22	11	22
1	62	0	T4N2M1	4	2	1	IVA	44	1.19	2.95	18.23	52.92	169.38	1	44	22	22	22	22
1	64	0	T3N2M0	3	2	0	III	33	9.29	3.68	32.8	52.9	140.12	1	33	22	22	11	22
1	57	0	T4N2M0	4	2	0	IIIB	33	2.11	1.41	91.9	52.87	99.95	1	44	22	22	11	22
1	57	1	T4N0M0	4	0	0	IIIA	33	3.37	3.94	17.52	52.73	122.30	1	44	22	11	11	22
1	53	0	T2N2M0	2	2	0	IIIA	33	2.09	0.76	21.25	49.1	127.54	1	22	22	22	11	22
0	50	0	T4N1M0	4	1	0	IIIA	33	12.14	2.64	18.63	48.71	154.93	1	44	22	22	11	22
0	58	0	T2N0M1	2	0	1	IV	44	14.16	1.66	17.43	48.6	134.28	1	22	22	11	22	22
0	67	0	T3N3M1	3	3	1	IVB	44	17.94	1.92	13.03	48.36	124.06	1	33	22	22	22	22
1	62	1	T3N2M1	3	2	1	IVa	44	3.53	9.82	24.85	48.06	128.24	1	33	22	22	22	22
1	66	0	T2N2M1	2	2	1	IVB	44	111.3	15.91	38.81	46.88	172.88	1	22	22	22	22	22
1	58	0	T4NXMX	4	x	x	IV	44	23.4	6.08	14.38	46.71	170.86	1	44	22			22
1	57	0	T1N0M1b	1	0	1b	IV	44	1.21	1.37	11.79	46.56	158.68	1	11	11	11	22	22
1	72	0	T4N2M1	4	2	1	IV	44	5.09	3.05	21.47	46.49	157.79	1	44	22	22	22	22
1	63	0	T3N0M0	3	0	0	IIB	22	52.4	10.73	28.74	46.41	106.82	1	33	22	11	11	11
1	73	1	T2N1M0	2	1	0	IIB	22	3.57	3.51	26.02	46.26	113.61	1	22	22	22	11	11
0	52	0	T4N3M1c	4	3	1c	IV	44	12.31	4.09	23.11	46.22	155.45	1	44	22	22	22	22
1	71	0	T1N0M0	1	0	0	IA	11	3.12	3.33	13.34	44.86	91.88	1	11	11	11	11	11
1	50	1	T1N0M0	1	0	0	I	11	5.38	1.83	17.36	44.66	80.65	1	11	11	11	11	11
1	53	0	T4N3M1c	4	3	1c	IVb	44	10.16	3.89	15.04	44.43	166.98	1	44	22	22	22	22
1	61	0	T2N2M0	2	2	0	IIIA	33	40.83	2.6	19.99	44.27	104.92	1	22	22	22	11	22
0	43	1	T2N3M0	2	3	0	IIIB	33	1.47	1.1	12.49	42.7	174.41	1	22	22	22	11	22
1	39	0	T4N3M1	4	3	1	IVb	44	2.58	1.46	12.37	42.41	146.73	1	44	22	22	22	22
1	76	1	T1N3M0	1	3	0	IIIB	33	2.74	5.59	19.75	42.38	161.41	1	11	11	22	11	22
0	51	0	T2N0M0	2	0	0	IB	11	0.77	1.38	19.03	41.64	174.38	1	22	22	11	11	11
1	74	1	T4N3M0	4	3	0	IIIC	33	6.31	61.25	63.61	41.09	109.98	1	44	22	22	11	22
0	51	0	T4N2M1	4	2	1	IV	44	0.96	2.08	16.75	40.86	161.95	1	44	22	22	22	22
1	55	0	T3N2M1	3	2	1	IVB	44	71.14	4.56	18.48	40	133.52	1	33	22	22	22	22
1	71	0	T4N3M1c	4	3	1c	IVB	44	16.28	11.11	34.2	39.44	169.22	1	44	22	22	22	22
0	46	0	T4N3M1	4	3	1	IVB	44	7.14	12.76	44.98	39.3	151.32	1	44	22	22	22	22
1	74	1	T4N2M0	4	2	0	IIIb	33	3.28	1.23	27.38	38.93	151.51	1	44	22	22	11	22
1	53	1	T2N3M0	2	3	0	IIIb	33	2.26	2.16	16.63	38.9	132.35	1	22	22	22	11	22
0	50	0	T2N2M1	2	2	1	IVA	44	4.83	1.52	19.27	38.16	146.27	1	22	22	22	22	22
0	63	0	T4N1M1	4	1	1	III	33	16.96	2	22.69	37.86	166.45	1	44	22	22	22	22
1	53	0	T2N2M1	2	2	1	IV	44	22.17	10.16	36.01	37.54	84.90	1	22	22	22	22	22
1	70	0	TxN0M1a	x	0	1a	IIIA	33	5.63	4.16	14.22	37.27	173.48	1	XX		11	22	22
0	63	0	T1N0M0	1	0	0	IB	11	0.52	1.55	15.29	37.03	166.61	1	11	11	11	11	11
1	68	0	T1N0M0	1	0	0	IA	11	4.14	1.41	22.23	36.87	156.64	1	11	11	11	11	11
1	55	0	T2N3M1c	2	3	1c	IVB	44	4.99	4.02	18.2	36.86	167.24	1	22	22	22	22	22
1	51	0	T2N2M1b	2	2	1b	IVA	44	1.45	2.05	38.75	36.84	174.24	1	22	22	22	22	22
1	63	1	T4N3M0	4	3	0	IIIc	33	2.93	52.58	27.44	35.55	100.63	1	44	22	22	11	22
0	55	0	T2N0M0	2	0	0	IB	11	1.35	1.53	13.41	35.22	90.29	1	22	22	11	11	11
0	66	0	T2N0M1c	2	0	1c	IVB	44	23.2	2.34	17.36	34.36	171.89	1	22	22	11	22	22
0	46	0	T3N3M0	3	3	0	III	33	20.21	1.8	16.59	33.89	125.62	1	33	22	22	11	22
1	72	0	T2N2M0	2	2	0	IIIA	33	4.26	4.03	16.27	32.89	137.57	1	22	22	22	11	22
0	48	0	T4N2M1	4	2	1	IVA	44	60.95	0.96	18.32	32.19	158.93	1	44	22	22	22	22
0	43	1	T2N3M0	2	3	0	IIIB	33	0.96	2.6	29.41	31.98	174.28	1	22	22	22	11	22
1	67	1	T4NXM1b	4	x	1b	IVA	44	3.02	1.97	17.92	31.73	173.98	1	44	22		22	22
1	60	1	T2N0M0	2	0	0	IIA	22	5.34	5.87	18.17	31.47	137.86	1	22	22	11	11	11
1	63	0	T4N2M1c	4	2	1c	IVB	44	2.91	2.36	24.42	30.87	151.92	1	44	22	22	22	22
1	53	0	T3NXM1b	3	x	1b	IVA	44	1.98	1.83	21.02	30.87	161.67	1	33	22		22	22
1	58	1	T3N3M1	3	3	1	IVB	44	3.13	1.81	12.63	30.01	123.33	1	33	22	22	22	22
1	72	0	T2N2M0	2	2	0	III	33	4.36	4.13	33.2	29.95	140.49	1	22	22	22	11	22
1	62	0	T1bN3M1c	1b	3	1c	IVB	44	126.1	10.52	18.51	29.68	173.47	1	11	11	22	22	22
0	50	0	T3N2M0	3	2	0	IIIB	33	1.73	3.02	14.81	29.16	103.33	1	33	22	22	11	22
1	65	1	T4N2M1	4	2	1	IVB	44	3.91	8.44	30.78	28.89	160.36	1	44	22	22	22	22
0	51	0	T2N2M0	2	2	0	IIIA	33	1.78	1.52	67.36	28.83	152.78	1	22	22	22	11	22
0	54	0	T2N2M1	2	2	1	IVB	44	101.1	2.39	19.19	28.79	147.31	1	22	22	22	22	22
0	45	0	T3N3M0	3	3	0	IIIC	33	18.83	2.12	13.14	27.84	149.88	1	33	22	22	11	22
1	70	0	T2N3M1	2	3	1	III	33	2.87	2.22	25.22	27.77	163.31	1	22	22	22	22	22
1	55	1	T1N3M1	1	3	1	III	33	4.81	5.76	25.93	27.47	90.50	1	11	11	22	22	22
1	57	0	T3N3M1	3	3	1	IVA	44	104.7	25.95	19.5	23.68	167.24	1	33	22	22	22	22
0	74	0	T4N2M1	4	2	1	III	33	2.74	4.03	17.5	15.81	151.12	1	44	22	22	22	22

ler Male=1 Fema	Age	CEA	CYFRA	NSE	PROGRP	RRM2	Group
0	48	1.95	1.38	16.58	27.22	117.01	0
0	39	0.86	1.59	16.04	27.46	136.99	0
1	53	1.65	3.96	12.77	27.63	152.10	0
1	50	0.98	1.69	15.16	29.17	106.86	0
0	39	1.43	1.29	14.6	30.22	81.97	0
0	57	0.85	1.18	13.18	30.4	100.39	0
1	57	1.11	1.81	14.16	31.29	115.66	0
1	59	2.10	2.01	13.95	31.29	124.45	0
1	40	1.67	1.82	23.4	31.62	91.44	0
0	57	0.79	2.82	22.56	31.92	153.53	0
1	46	2.05	1.36	12.11	32.29	62.17	0
0	70	1.46	1.43	15.37	32.31	147.27	0
0	65	1.09	2.96	13.6	32.48	72.03	0
0	67	1.18	1.6	15.32	32.67	81.89	0
1	41	1.09	2.18	12.36	32.87	157.53	0
1	56	1.62	2.05	19.51	33.17	91.23	0
1	55	0.62	2.65	11.11	33.44	109.98	0
1	68	0.69	1.43	15.23	33.54	115.11	0
1	54	1.35	2.55	17.62	34.79	120.23	0
0	57	0.98	1.25	31.17	35.98	82.27	0
1	45	2.05	1.95	13.19	36.05	147.39	0
1	65	0.65	1.65	16.65	36.1	138.95	0
1	77	1.81	1.2	18.86	36.3	76.16	0
1	57	2.04	1.6	15.76	36.39	150.33	0
0	77	0.59	2.36	17.26	36.87	90.17	0
0	47	5.76	1.39	12.97	38.12	124.50	0
0	40	0.75	1.66	18.88	39.27	153.40	0
1	40	1.59	2.02	15.77	39.39	137.25	0
1	45	1.06	1.05	16.77	39.53	127.00	0
0	70	1.95	1.06	17.54	40.22	110.06	0
0	51	1.36	2.04	13.45	40.93	130.18	0
1	43	1.65	3.76	15.18	41.45	86.21	0
1	36	0.74	1.28	15.32	42.69	109.31	0
0	39	0.47	1.82	15.93	44.05	103.83	0
1	46	2.62	1.48	10.69	44.2	112.75	0
0	47	2.01	2.34	10.74	44.65	105.71	0
1	52	0.94	2.83	8.92	45.48	130.27	0
1	36	1.91	2.53	15.76	47.9	84.62	0
0	69	3.73	1.92	13.54	49.01	79.96	0
0	51	0.31	1.81	17.11	53.07	127.70	0
0	41	1.27	3.82	9.93	53.99	132.65	0
1	48	2.79	1.3	13.31	55.27	88.38	0
1	55	0.83	2.25	13.62	55.77	127.70	0
0	49	2.02	2.16	13.32	56.16	110.80	0
1	44	1.27	1.36	15.81	62.92	150.66	0
0	57	3.04	3.12	15.16	66.4	95.32	0
1	57	1.39	2.12	13.81	66.5	147.64	0
1	54	2.61	1.93	18.26	76.29	106.90	0
1	61	1.05	1.93	9.39	77.88	86.71	0
0	69	0.46	1.36	9.49	82.53	87.80	0
