# Supplementary material for: Ribonucleotide reductase regulatory subunit M2 (RRM2) as a potential sero-diagnostic biomarker in non-small cell lung cancer
Source: PLoS One. 2023 Sep 12;18(9):e0291461. doi: 10.1371/journal.pone.0291461 (PMC10497127; doi:10.1371/journal.pone.0291461)
Supplement: Attachment — Submitted filename: COMMENTS FOR THE AUTHOR.doc [file pone.0291461.s006.doc]

COMMENTS FOR THE AUTHOR:

This study is interesting and provides a new target for non-small cell lung cancer, but it also has the following problems:

1、Please improve the language style, some sentences need to be more in line with English writing.
2、I would like to ask the author to improve on the introduction section, in which the author mentions the traditional markers and further mentions the progress and shortcomings of the new markers that are currently emerging, such as exosomes, circulating tumor DNA, non-coding RNA, etc.
3、In Materials and methods-Data resources. Due to involving human samples, please register the authors standardize clinical ethics, the Chinese clinical trial registration center - the world health organization (who) international clinical trial register platform registered institution ", the website: <https://www.chictr.org.cn/.>
4、"|log2 fold change (FC)| ≥ 2" value is 2, why not set to 1.
5、This paper mainly studies the markers of non-small cell lung cancer, which includes lung adenocarcinoma, lung squamous cell carcinoma and large cell carcinoma. The authors conclude that RRM2, UBE2C, MAD2L1, KIF20A, CDK1, and CCNB2 were statistically significant (P < 0.05). Are these genes also expressed in large cell carcinoma in the same way?
6、Please check the Data before and after. In the "Data resources" section, the blood sample is 100 examples. Why is it changed to 110 examples in the "Patient sample analysis." section?
7、Why does the "Validation and survival analysis of hub genes" part only analyze overall survival analysis, Without analyzing disease-free survival and Disease-specific survival?
8、"Tissue analysis:" this part of why the emergence of TNBC, study is not non-small cell lung cancer?
9、Please improve the discussion of this part, refer to the introduction.
