# Supplementary material for: Ribonucleotide reductase regulatory subunit M2 (RRM2) as a potential sero-diagnostic biomarker in non-small cell lung cancer
Source: PLoS One. 2023 Sep 12;18(9):e0291461. doi: 10.1371/journal.pone.0291461 (PMC10497127; doi:10.1371/journal.pone.0291461)
Supplement: Attachment — Submitted filename: reviewer.doc [file pone.0291461.s008.doc]

Thank you very much for your revision. Now I still have the following questions:

1、Why is the annotation not modified in the manuscript, and the color is not prominent in the modified area? I don't know what specific content you modified?
2、EVOlyzer-2 150 platform. What is this?
