# Supplementary material for: Ribonucleotide reductase regulatory subunit M2 (RRM2) as a potential sero-diagnostic biomarker in non-small cell lung cancer
Source: PLoS One. 2023 Sep 12;18(9):e0291461. doi: 10.1371/journal.pone.0291461 (PMC10497127; doi:10.1371/journal.pone.0291461)
Supplement: Attachment — Submitted filename: Response to Reviewers.docx [file pone.0291461.s009.docx]

**Response Letter**

Dear reviewers:

Thank you for your careful review of our manuscript and your valuable comments concerning our manuscript (ID: PONE-D-23-12628R1).

On the basis of the advices raised by reviewers, the manuscript was revised thoroughly after the discussion and approval by all the authors. The main corrections in the paper and response to the reviewers are summarized below.

We would like to express our great appreciation for your suggestions and comments. It is valuable and very helpful for revising and improving our paper, as well as the important guiding significance to our researches.

Should you have any questions, please feel free to contact us at xiumingzhai@163.com.

Looking forward to hearing from you.

Yours sincerely,

Xiuming Zhai

Department of Laboratory Medicine,

Chongqing Traditional Chinese Medicine Hospital

No. 6, Panxi Qizhi Road, 400021, Chongqing, P.R China

Tel.: (+86) 023-67063765

**Response to Reviewer #1:**

***1. Why is the annotation not modified in the manuscript, and the color is not prominent in the modified area? I don't know what specific content you modified?***

Response: Thank you for your careful and patient consideration of my manuscript. Please allow me to explain your confusion.

(1) I can only see your comments in the PLOS ONE submission system, but I do not have access to your annotated manuscript. After communicating with the PLOS ONE editor several times, I have still not been able to access your annotated manuscript. However, we have carefully revised the linguistic aspects of the revised manuscript again, based on your latest suggestions.

(2) As the PLOS ONE submission system requires the author to upload "Revised Manuscript with Track Changes.docx" and "Manuscript -ClearCopy.docx", I can't mark up the changes again in the "Manuscript -ClearCopy.docx". I've now marked this revision in red and underlined the previous revision in "Revised Manuscript with Track Changes.docx".

Thank you again for your valuable time in helping our team improve academically.

***2. EVOlyzer-2 150 platform. What is this?***

Response: Thank you for your consideration. The Freedom EVOlyzer is a fully automated enzyme immunoassay instrument and the 150 is a model of this instrument.
